# Systematic Analysis of the Cytokine and Anhedonia Response to Peripheral Lipopolysaccharide Administration in Rats

**DOI:** 10.1155/2016/9085273

**Published:** 2016-07-18

**Authors:** Steven Biesmans, Liam J. R. Matthews, Jan A. Bouwknecht, Patrick De Haes, Niels Hellings, Theo F. Meert, Rony Nuydens, Luc Ver Donck

**Affiliations:** ^1^BIOMED, Hasselt University, Agoralaan C Building, 3590 Diepenbeek, Belgium; ^2^Neuroscience, Janssen Research & Development, Division of Janssen Pharmaceutica NV, Turnhoutseweg 30, 2340 Beerse, Belgium

## Abstract

Inflammatory processes may cause depression in subsets of vulnerable individuals. Inflammation-associated behavioral changes are commonly modelled in rodents by administration of bacterial lipopolysaccharide (LPS). However, the time frame in which immune activation and depressive-like behavior occur is not very clear. In this study, we showed that systemic administration of LPS robustly increased circulating levels of corticosterone, leptin, pro- and anti-inflammatory cytokines, and chemokines. Serum concentrations of most analytes peaked within the first 6 h after LPS injection and returned to baseline values by 24 h. Chemokine levels, however, remained elevated for up to 96 h. Using an optimized sucrose preference test (SPT) we showed that sickness behavior was present from 2 to 24 h. LPS-induced anhedonia, as measured by decreased sucrose preference, lasted up to 96 h. To mimic the human situation, where depression develops after chronic inflammation, rats were preexposed to repeated LPS administration or subchronic restraint stress and subsequently challenged with LPS. While these procedures did not increase the duration of anhedonia, our results do indicate that inflammation may cause depressive symptoms such as anhedonia. Using our SPT protocol, more elaborate rodent models can be developed to study the mechanisms underlying inflammation-associated depression in humans.

## 1. Introduction

Major depressive disorder, or depression, is a serious medical illness with a life time prevalence of around 16% [1]. It is predicted that by 2030 depression will be the second leading cause of disability worldwide [2]. Clinical manifestations of depression include a range of symptoms, such as depressed mood, anhedonia (inability to experience pleasure from naturally rewarding activities), feelings of worthlessness or excessive guilt, decreased appetite and weight, fatigue, and recurrent suicidal ideations [3]. For many years, pharmacological research in depression has been focused on the monoamine theory, which proposes that depression is caused by decreased monoamine function in the brain and that drugs which correct this deficit, for example, selective serotonin reuptake inhibitors (SSRIs) and serotonin-norepinephrine reuptake inhibitors (SNRIs), can treat the disorder [4, 5]. Though the monoamine systems are clearly involved in the etiology of depression, it is now generally accepted that a more complex interplay between genetics and environmental factors underlies its pathophysiology. Findings from clinical studies indicate that inflammatory processes might also be involved in the pathogenesis of depression, at least in a subset of susceptible individuals (for reviews see [6–10]).

Based on these observations, several rodent models of inflammation-associated depression have been developed. One of the most used models involves administration of bacterial lipopolysaccharide (LPS), which is a potent activator of the immune system. Behavioral studies in rodents have shown that systemic LPS injection induces a sickness response, characterized by hypolocomotion, social withdrawal, fatigue, anorexia, and alterations in sleep patterns and cognition [6]. There are some indications that LPS-induced sickness is followed by a depressive-like phenotype in which rodents display behavior similar to clinically relevant symptoms of depression in humans [11–13]. However, the time frame in which potential depressive-like behavior occurs relative to sickness is not clear and findings often vary across labs. Some studies indicate that depressive-like behavior can be observed in the absence of sickness 24 h after systemic LPS administration [11–13], while others report that at this time sickness is still present and hence confounds measurements of depressive-like behavior [14–17]. Indeed, sick animals display reduced locomotor activity, which can confound measurements of immobility used to estimate behavioral despair in paradigms such as the forced swim test [6]. Moreover, sick animals show reduced eating and drinking behavior, thereby potentially confounding measures of sweetened fluid intake in paradigms designed to evaluate anhedonia. Therefore, it is of crucial importance to include measures of sickness when assessing depressive-like behavior.

In a previous study we characterized behavioral changes induced by systemic LPS injection in mice [17]. This work showed that the time course of sickness and anhedonia can be evaluated by measuring total volume intake and sucrose preference in an optimized sucrose preference test (SPT). To extend our previous work, we characterized sickness and the anhedonic response to systemic LPS injection using a SPT in rats. First, the dose dependency of LPS-induced behavioral changes during the first 24 h after LPS administration was evaluated across a panel of behavioral assays. After identifying a dose that induced potential anhedonia, the immunological response to systemic LPS was measured by quantifying serum levels of corticosterone, leptin, and a selection of cytokines and chemokines over time. Then, the time course of sickness and the anhedonic response to systemic LPS was assessed using an optimized SPT. Finally, in an attempt to mimic the chronic nature of depression in humans more carefully, our SPT protocol was used to test whether preexposure to repeated LPS administration or subchronic stress influenced the anhedonic response to a subsequent LPS challenge.

## 2. Methods

### 2.1. Animals and LPS

All studies were conducted in male Sprague-Dawley rats (Harlan, Netherlands) weighing 200–220 g on arrival. Animals were housed in groups of 4 in plexiglass individually ventilated cages (*L* × *W* × *H*: 43 × 32 × 18 cm; Tecniplast, Italy) for one week to acclimate prior to experiments. Procedure rooms were maintained at a temperature of 22 ± 2°C and a humidity of 54 ± 2%, with a 12 : 12 h light-dark cycle (lights on at 06:00 a.m. with a 30-minute sunrise and dusk phase). Unless mentioned otherwise, food and water were available* ad libitum*. All experimental protocols were approved by the Institutional Ethical Committee on Animal Experimentation, in compliance with Belgian law (Royal Decree on the protection of laboratory animals, April 6, 2010) and conducted in facilities accredited by the Association for the Assessment and Accreditation of Laboratory Animal Care (AAALAC).

Lipopolysaccharide (LPS) from* Escherichia coli* (serotype 055:B5) was purchased from Sigma-Aldrich and freshly dissolved in sterile saline prior to injection.

### 2.2. Serum Collection

To measure the effect of peripheral LPS administration on serum levels of a selection of analytes, rats were injected i.p. with either vehicle or 0.63 mg/kg LPS (*n* = 12 per group). Just before and at 1 h, 2 h, 6 h, 24 h, 48 h, 72 h, and 96 h after the immune challenge, rats were briefly anesthetized by inhalation of 2% isoflurane, blood was collected from the tail artery, and the rats were returned to their home cage. Serum was obtained by keeping the blood samples in Vacutainer SST II Advance blood tubes (BD Biosciences, product ID 367955) for 30 minutes at room temperature. Then the samples were centrifuged (1300 ×g, 10 min, room temperature), aliquoted, flash frozen in liquid nitrogen, and stored at −80°C until analysis.

### 2.3. Cytokine Measurements

Serum levels of chemokine (C-X-C motif) ligand 1 (CXCL1), interferon-*γ* (IFN-*γ*), interleukin- (IL-) 1*β*, IL-6, IL-10, leptin, monocyte chemoattractant protein-1 (MCP-1), macrophage inflammatory protein-1*α* (MIP-1*α*), and tumor necrosis factor-*α* (TNF-*α*) were simultaneously determined using a rat cytokine/chemokine magnetic bead panel kit from Merck Millipore. This assay is based on Luminex*™* technology in which magnetic beads with a distinct emitting fluorescence pattern are coated onto antibodies that specifically capture individual cytokines. All steps in the assay were conducted according to the manufacturer's instructions. A Bio-Plex 200 System (Bio-Rad) was used to measure the fluorescent signal and the data was analyzed using Bio-Plex Manager 5.0 software (Bio-Rad) with five-parameter logistic regression curve fitting. Cytokine levels below detection limit were assigned a value equal to the lowest detectable value of that cytokine. Cytokine values outside of the average + 3 times standard deviation range were considered outliers and were excluded from all calculations. This was the case for less than 2% of all measured cytokines.

### 2.4. Corticosterone Measurements

Serum concentrations of corticosterone were determined using a commercial ELISA kit supplied by Enzo Life Sciences (Product ID 900-097). All procedures were carried out as per manufacturer's instructions.

### 2.5. Behavioral Tests

The open field test (OFT) and forced swim test (FST) setups were custom-made. In these paradigms, groups of naive rats (*n* = 12 per group) were injected i.p. with 0, 0.31, 0.63, or 1.25 mg/kg LPS and tested at 2 h, 6 h, or 24 h after LPS administration. This dose range of LPS was selected based on results from our previous experiments in mice [17].

#### 2.5.1. Open Field Test

The OFT setup consisted of a circular arena with a diameter of 1.2 m. A video camera with an infrared filter was fixed 1 m above the arena and infrared illumination was provided from the bottom for optimal detection and tracking of the rats. This setup allowed the testing of one rat at a time. Exactly 2 sec after detection of a rat, tracking was started and performed for 10 min using EthoVision 6.1 software (Noldus, Netherlands), set up to detect immobility time and distance moved. In this test, exploratory behavior by the animal was used to measure locomotor activity.

#### 2.5.2. Forced Swim Test

The FST setup consisted of four independent plexiglass cylinders (diameter 19 cm) which were flushed and filled with water (30 cm deep, 24-25 degrees Celsius). The four cylinders allowed testing of four rats per session. A video camera with an infrared filter was fixed onto a frame in front of each cylinder and infrared illumination was provided to allow optimal detection and tracking of the rats. Exactly 2 sec after detection of each individual rat, tracking was started and performed using EthoVision 6.1 software (Noldus, Netherlands). Each FST test consisted of two sessions: a 15 min training session on the day before LPS administration and a 6 min test session at the relevant time after LPS. Immobility time and distance moved (based on center point of gravity of the detected surface) were measured during each session and the duration of immobility was evaluated as a measure of behavioral despair.

#### 2.5.3. Sucrose Preference Test in Fluid Deprived Rats

Animals were single-housed in individually ventilated cages (*L* × *W* × *H*: 35 × 31 × 16 cm; Tecniplast, Italy) fitted with two 250 mL drinking bottles and* ad libitum* access to food. Each of the drinking bottles contained either filtered tap water or a 1% sucrose solution. The location of each bottle on the cage was randomized every day, with half the animals receiving sucrose on the left and the other half on the right. Prior to LPS administration, rats were familiarized to the sucrose solution by presenting them with water/water (W/W) or water/sucrose (W/S) for 24 h each on 2 consecutive days. Then, the rats were fluid deprived overnight and injected i.p. with 0, 0.31, 0.63, or 1.25 mg/kg LPS (*n* = 12 per group). At 2 h, 6 h, and 24 h after LPS administration all rats were exposed to preweighed drinking bottles containing W/S. After 1 h the bottles were removed and weighed using Software Wedge for Windows 1.2 (TAL Technologies).

#### 2.5.4. Sucrose Preference Test in Undeprived Rats

**Figure 1:**
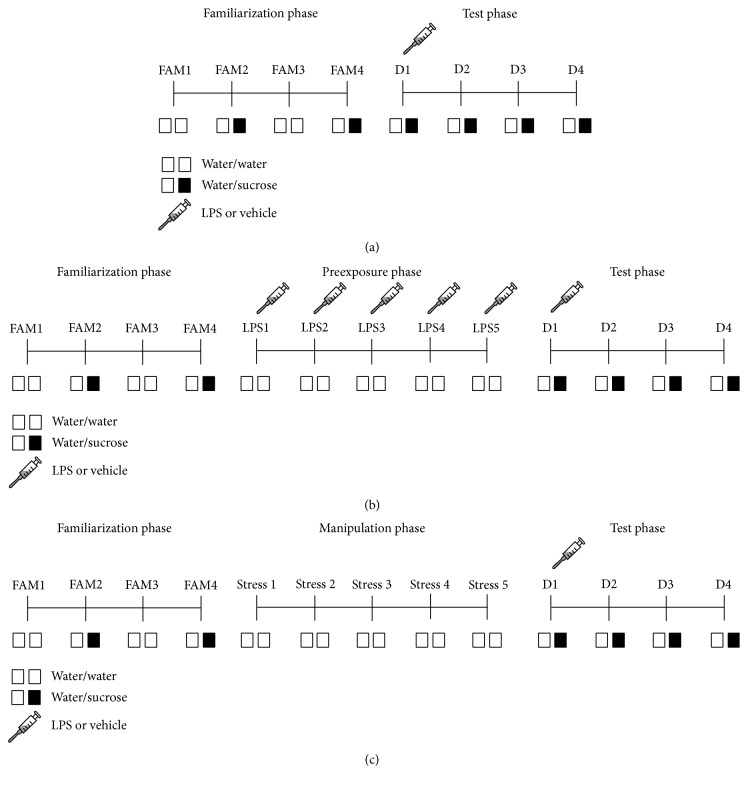
Experimental design of the sucrose preference tests performed using fluid undeprived rats. In the* familiarization phase*, rats were presented for 24 h with two water-filled bottles (W/W) on familiarization day 1 (FAM1) and FAM3, or one water- and one 1% sucrose-filled bottle (W/S) on FAM2 and FAM4. In all studies, the* test phase* started by injecting the rats with LPS (0.63 mg/kg, i.p.) or vehicle. Voluntary consumption of water and sucrose was measured during a period of 24 h for 4 days (D1–D4) in the* test phase* (a–c). The effect of repeated systemic LPS injection on anhedonia was evaluated by preceding the* test phase* by a* preexposure phase* during which rats received a daily i.p. injection of 0.63 mg/kg LPS or vehicle for 5 consecutive days (LPS1–LPS5) (b). To assess the effect of stress on LPS-induced anhedonia, the* test phase* was preceded by a* manipulation phase* during which rats were subjected to 1 h of restraint stress daily for 5 consecutive days (Stress 1–Stress 5) (c).

All of the remaining SPT experiments started with a 4-day* familiarization phase*, during which the rats were presented for 24 h with two water-filled bottles (W/W) on familiarization day 1 (FAM1) and FAM3 or one water- and one 1% sucrose-filled bottle (W/S) on FAM2 and FAM4 (Figure 1(a)). The bottles were removed between 08:00 and 09:00 a.m. each day and weighed using Software Wedge for Windows 1.2. Then, the animals were weighed and freshly prepared bottles were put onto the cages.

To assess the effect of a single peripheral bolus of LPS on anhedonia over time, the* test phase* started by weighing and injecting rats with either vehicle or 0.63 mg/kg i.p. LPS (*n* = 12 per group). Immediately after LPS administration, the rats were put into their home cage and given access to W/S for 4 consecutive days. This experiment was repeated three times using 12 naive animals per treatment group in each trial and the data was pooled prior to analysis so that the final *n* = 36 per group.

The effect of repeated systemic LPS injection on anhedonia was evaluated by randomizing rats across 4 experimental groups, that is, 5 days of vehicle + vehicle on test day (5 Veh + Veh), 5 days of vehicle + LPS on test day (5 Veh + LPS), 5 days of LPS + vehicle on test day (5 LPS + Veh), and 5 days of LPS + LPS on test day (5 LPS + LPS) (*n* = 12 per group). After the* familiarization phase*, a* preexposure phase* was introduced in which rats from the 5 days of vehicle groups were injected i.p. with vehicle while rats from the 5 days of LPS groups received a daily i.p. injection of 0.63 mg/kg LPS for 5 consecutive days (Figure 1(b)). All rats had* ad libitum* access to food and water during the* preexposure phase*. Three days after the last LPS administration, rats were injected with an acute bolus of either vehicle or 0.63 mg/kg i.p. LPS and presented with W/S for 24 h for 4 consecutive days.

To assess the effect of stress on LPS-induced anhedonia, rats were randomized across 4 experimental groups, that is, no stress + vehicle (NS + Veh), no stress + LPS (NS + LPS), stress + vehicle (S + Veh), and stress + LPS (S + LPS) (*n* = 12 per group), and a* manipulation phase* was introduced in between the* familiarization phase* and* the test phase* (Figure 1(c)). During this* manipulation phase*, animals in the stress groups were weighed and subjected to 1 h of physical restraint per day using a transparent rat restrainer (*D* × *H*: 5 × 23 cm; length adjusted to tightly enclose the rat) for 5 consecutive days. To control for possible effects of handling stress, rats from the no stress groups were weighed, handled, and put back in their home cage. All rats had* ad libitum* access to food and water during the* manipulation phase*. The* test phase* started 3 days after the* manipulation phase* by injecting the rats i.p. with either vehicle or 0.63 mg/kg LPS. Immediately after LPS administration, all animals were presented with W/S for 4 consecutive days.

#### 2.5.5. Evaluation of Parameters in Sucrose Preference Test

In all SPT experiments, the amount drunk by a rat was determined by subtracting the weight of a bottle at the start of the observation period and at the end (taking fluid density as 1 g/mL). Total fluid intake was taken as the total change in volume from both bottles combined, while sucrose preference was calculated as a percentage of consumed sucrose solution of the total fluid intake. A total fluid intake greater than the mean + two times standard deviation was considered to be an invalid measure that probably resulted from leaking bottles. Invalid measures were replaced by the mean of all the bottles either on the relevant side (for W/W) or for either water or sucrose (for W/S). This happened for less than 2% of bottle measurements in any given experiment. Total volume intake was evaluated as a primary measure for sickness behavior (reduction versus normal daily intake), while sucrose preference was used as a measure of anhedonia. Change in body weight was calculated by subtracting the weight at a given time point from the weight at the start of the experiment. These time points are specified for each experiment in the Results.

### 2.6. Statistical Analysis

Data were analyzed using SPSS Statistics software (Version 20 for Windows, IBM Inc). Analysis of variance (ANOVA) or repeated measure ANOVA (rmANOVA) was performed to assess the statistical significance of differences across treatment groups. A Greenhouse-Geisser correction epsilon (*ε*) was used in case of repeated measures analysis to correct for potential violation of the sphericity assumption [18]. This correction multiplies both the numerator and the denominator degrees of freedom by epsilon and the significance of the *F*-ratio is evaluated with the new degrees of freedom, resulting in a more conservative statistical test. To account for the skewness of the data distribution, concentrations of serum analytes were log-transformed prior to analysis. Significance was accepted for the ANOVAs and rmANOVAs when *p* < 0.05. When appropriate, post hoc comparisons were made by using an independent samples *t*-test with a Bonferroni-corrected *p* value. For consistency between the analysis and the visualization of serum analyte concentrations, the group means and its standard error of the mean (SEM) were back-transformed and visually presented on a logarithmic scale. All other data are expressed as mean ± SEM on a linear scale.

## 3. Results

### 3.1. Systemic LPS Administration Causes Sickness and Anhedonia in a Dose- and Time-Dependent Manner

**Figure 2:**
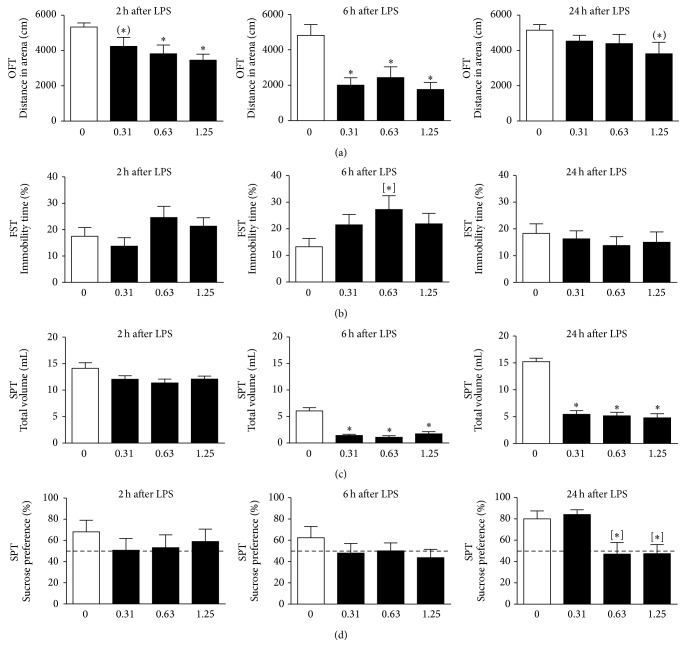
Systemic LPS administration causes sickness and anhedonia in a dose- and time-dependent manner. Intraperitoneal LPS injection induced sickness behavior, as seen by reduced locomotor activity in the open field test (OFT) (a) and decreased total volume intake in the sucrose preference test (SPT) (c). At 24 h after administration, a dose of 0.63 and 1.25 mg/kg LPS reduced sucrose preference (d), thereby potentially indicating development of anhedonia. However, a single i.p. injection of LPS did not induce clear depressive-like behavior in the forced swim test (FST) (b). Please note that in the OFT and FST naive animals were used at all time points, whereas in the SPT rats were tested repeatedly. Dashed lines indicate chance level for sucrose preference. Graphs are plotted as mean + SEM (*n* = 12 per group). OFT and FST data were analyzed by multivariate ANOVA, SPT data by rmANOVA, and followed by independent samples *t*-test. ^(*∗*)^0.1 < *p* < 0.05; ^*∗*^ *p* < 0.05 compared to 0 mg/kg LPS group; ^[*∗*]^ *p* < 0.05 compared to 0 mg/kg LPS group in absence of rmANOVA effects.

The total distance travelled in the OFT is a general measure for exploration and can be used as a marker of sickness behavior. Factorial ANOVA revealed a main effect of LPS dose (*F*(3,110) = 11.1, *p* < 0.001) and time point (*F*(2,110) = 15.6, *p* < 0.001) for total distance travelled. Post hoc analysis indicated that systemic LPS administration reduced locomotor activity in a dose-dependent manner at 2 h (Figure 2(a)). This LPS-induced reduction in exploration was more pronounced at 6 h after LPS but disappeared at 24 h.

The effect of systemic LPS administration on behavioral despair was evaluated in the FST by placing the rats in a water-filled cylinder from which they cannot escape and measuring the time they remained immobile. Factorial ANOVA demonstrated that there was no main effect of LPS dose or time point tested. Explorative post hoc analysis indicated that rats injected with 0.63 mg/kg LPS showed a potential increase in immobility time at 6 h after administration (Figure 2(b)). Such an immobility response was not observed at any of the other time points or LPS doses used.

In the sucrose preference paradigm, sickness is evaluated by measuring the total volume of fluid an animal consumes during a predefined observation period, while sucrose preference is used as marker for anhedonia. rmANOVA revealed a significant time × LPS dose interaction for total volume intake (*F*(6,80) = 12.3, *p* < 0.001, *ε* = 0.98). Post hoc analysis indicated that LPS reduced total volume intake at 6 h and 24 h to a similar extent at all doses (Figure 2(c)), suggesting suppression of drinking as a consequence of sickness. No main effect of time or LPS dose was found for sucrose preference. However, explorative post hoc analysis demonstrated that, at 24 h, sucrose preference was significantly reduced in rats that were injected with 0.63 or 1.25 mg/kg LPS (Figure 2(d)). Rats injected with 0.31 mg/kg LPS did not show reduced sucrose preference, while at this time they drank much less than vehicle-treated rats. This suggests that LPS-induced anhedonia is potentially detectable at a dose of 0.63 mg/kg and higher.

### 3.2. Systemic LPS Increases Serum Levels of Corticosterone, Cytokines, and Chemokines in a Time-Dependent Manner

**Figure 3:**
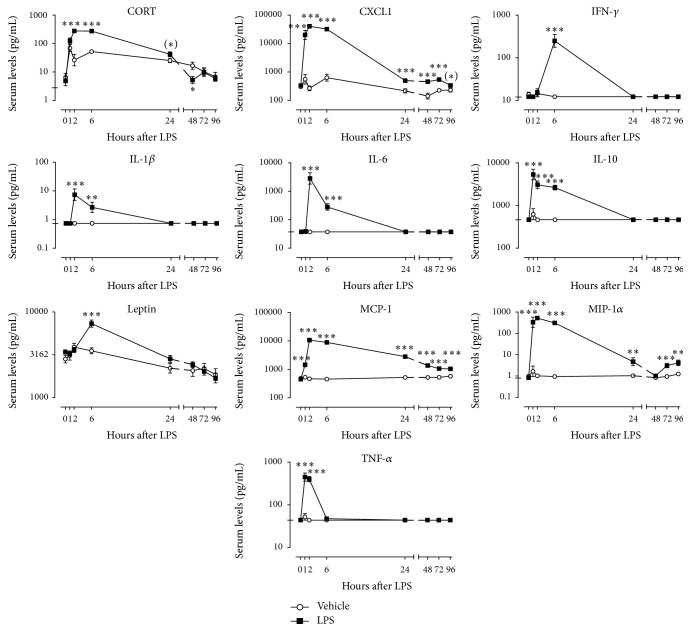
Peripheral LPS administration transiently increases serum levels of corticosterone, leptin, cytokines, and chemokines. Time curves of corticosterone (CORT), chemokine (C-X-C motif) ligand 1 (CXCL1), interferon-*γ* (IFN-*γ*), interleukin- (IL-) 1*β*, IL-6, IL-10, leptin, monocyte chemoattractant protein-1 (MCP-1), macrophage inflammatory protein-1*α* (MIP-1*α*), and tumor necrosis factor-*α* (TNF-*α*) quantified in serum at 0 h, 1 h, 2 h, 6 h, 24 h, 48 h, 72 h, and 96 h after LPS injection (0.63 mg/kg, i.p.). The detection limit of each analyte is indicated by a tick on the *y*-axis of its individual graph. Detection limits that fall below the lowest value on the *y*-axis are not presented. Graphs are plotted as mean ± SEM (*n* = 12 per group). Data were analyzed by rmANOVA followed by independent samples *t*-test. ^(*∗*)^0.1 < *p* < 0.05, ^*∗*^ *p* < 0.05, ^*∗∗*^ *p* < 0.01, and ^*∗∗∗*^ *p* < 0.001 compared to vehicle.

Based on the strong behavioral effects of 0.63 mg/kg LPS, it was decided to analyze the effect of this particular LPS dose on the release of a selection of hormones and cytokines in serum over time. Factorial rmANOVA revealed a significant time × LPS interaction for the analytes corticosterone (*F*(7,140) = 11.2, *p* < 0.001, *ε* = 0.47), CXCL1 (*F*(7,133) = 56.7, *p* < 0.001, *ε* = 0.30), IFN-*γ* (*F*(7,140) = 39.8, *p* < 0.001, *ε* = 0.29), IL-1*β* (*F*(7,140) = 14.9, *p* < 0.001, *ε* = 0.28), IL-6 (*F*(7,140) = 76.6, *p* < 0.001, *ε* = 0.19), IL-10 (*F*(7,140) = 35.1, *p* < 0.001, *ε* = 0.22), leptin (*F*(7,140) = 6.5, *p* < 0.001, *ε* = 0.53), MCP-1 (*F*(7,140) = 288.4, *p* < 0.001, *ε* = 0.38), MIP-1*α* (*F*(7,140) = 51.1, *p* < 0.001, *ε* = 0.33), and TNF-*α* (*F*(7,140) = 68.2, *p* < 0.001, *ε* = 0.23). Post hoc analysis showed that serum levels of corticosterone were elevated at 2 h, 6 h, and 24 h and fell below control values at 48 h after LPS administration (Figure 3). Furthermore, LPS caused a strong but short-lasting increase in the serum concentrations of most cytokines. Interestingly, the peak of this release did not occur at the same time for all cytokines. IL-10 and TNF-*α* peaked at 1 h, while CXCL1, IL-1*β*, IL-6, MCP-1, and MIP-1 reached their peak release at 2 h after LPS administration. IFN-*γ* and leptin were the only analytes that peaked at 6 h after LPS. Apart from the chemokines CXCL1, MCP-1, and MIP-1*α* all immune molecules had returned to control levels by 24 h.

### 3.3. LPS-Induced Anhedonia Can Be Measured in the Sucrose Preference Test

**Figure 4:**
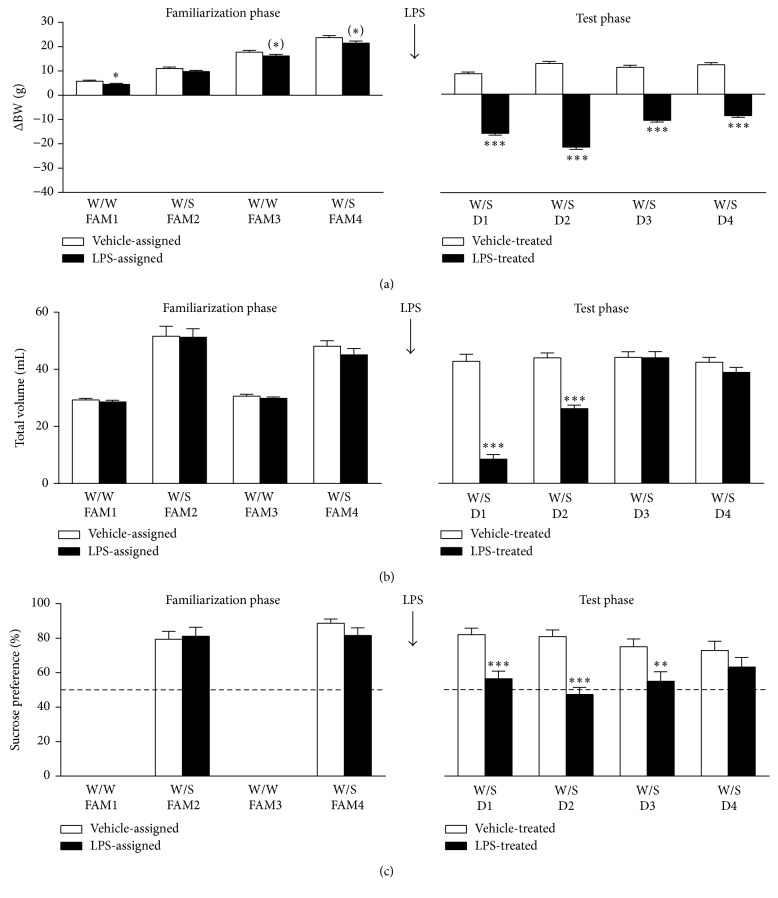
Systemic LPS injection reduces body weight, fluid intake, and sucrose preference in the sucrose preference test. An optimized sucrose preference test was used to evaluate the anhedonic response to LPS. During the* familiarization phase* of the experiment ((a–c) left panels), rats were exposed to 2 bottles of water (W/W) on familiarization day 1 (FAM1) and FAM3, while on FAM2 and FAM4 one bottle was filled with water and the other bottle contained a 1% sucrose solution (W/S). Three days after the* familiarization phase*, rats were injected i.p. with 0.63 mg/kg LPS or vehicle and voluntary consumption of water and sucrose was measured during a period of 24 h for 4 days (D1–D4) in the* test phase* ((a–c) right panels). Note that the growth rate of rats during each day of the* familiarization phase* was evaluated by calculating the body weight change (ΔBW) against their weight at the first day of the* familiarization phase* ((a) left panel). Growth rate in the* test phase* is presented as the body weight change at each day compared to the rats' weight right before LPS administration. Dashed lines in (c) indicate chance level for sucrose preference. Graphs are plotted as mean + SEM and represent pooled data from 3 separate but identical studies using 12 naive animals per treatment group in each experiment (total *n* = 36 per treatment group). Data were analyzed by rmANOVA followed by independent samples *t*-test. ^(*∗*)^0.1 < *p* < 0.05, ^*∗*^ *p* < 0.05, ^*∗∗*^ *p* < 0.01, and ^*∗∗∗*^ *p* < 0.001 compared to vehicle.

Based on the results from the first SPT study (Figure 2) and the time course of LPS-induced cytokine and chemokine release (Figure 3), an extended SPT study was performed to analyze the effects of peripheral LPS administration over a longer period of time. In this optimized experimental design, undeprived rats were subjected to a* familiarization phase* and a* test phase*. During the* familiarization phase* normal daily intake volume was assessed, animals were familiarized with exposure to sucrose, and a stable sucrose preference baseline was obtained. The growth rate of rats during each day of the* familiarization phase *was evaluated by calculating the body weight change against their weight at the first day of the* familiarization phase*. Factorial rmANOVA showed that there was a main effect of time (*F*(3,210) = 563.2, *p* < 0.001, *ε* = 0.69) and LPS assignment (*F*(1,70) = 5.1, *p* < 0.05, *ε* = 0.69) for change in body weight during the* familiarization phase*. Post hoc analysis indicated that rats in the LPS group had a statistically significant lower change in body weight at familiarization day 1 (FAM1) as compared to animals in the vehicle group (Figure 4(a), left panel). However, this difference was very small and can be considered as not biologically relevant. Rats from both groups continuously grew about 5 g per day throughout the* familiarization phase*, regardless of exposure type (W/W versus W/S). This indicates that the caloric value of sucrose did not influence the change in body weight.

For total volume intake during the* familiarization phase*, there was a flavor × repeat interaction (*F*(1,90) = 8.9, *p* < 0.01) but no effect of LPS assignment. Post hoc analysis indicated that the total volume intake increased substantially on days that rats were exposed to W/S when compared to W/W days. This increase was slightly reduced upon retesting (i.e., FAM4 versus FAM2) (Figure 4(b), left panel).

There were no time or group effects on sucrose preference during the* familiarization phase* and the rats showed a stable sucrose preference of around 80% on both W/S days (Figure 4(c), left panel).

In the* test phase*, the effect of systemic LPS on change in body weight, total daily intake volume, and sucrose preference was assessed over time. The growth rate of rats during each day of the* test phase* was evaluated by calculating the body weight change against their weight right before LPS administration. Factorial rmANOVA revealed a strong time × LPS interaction (*F*(3,210) = 86.2, *p* < 0.001, *ε* = 0.83) for change in body weight during the* test phase*. Post hoc analysis showed that systemic LPS injection reduced weight during the first 2 days after injection (D1 and D2) and that this weight decrease remained statistically significant throughout the* test phase* (Figure 4(a), right panel).

For total volume intake during the* test phase*, there was a time × LPS interaction (*F*(3,210) = 50.0, *p* < 0.001, *ε* = 0.87). In the first 24 h after administration (D1), LPS reduced total volume intake to less than one-third of the normal daily water intake, suggesting suppression of drinking as a consequence of sickness (Figure 4(b), right panel). On D2, LPS-injected rats still drank significantly less than rats that received vehicle, but their total volume intake was no longer lower than the normal daily water intake, thereby indicating that sickness had dissipated. No differences in total volume intake were found on D3 and D4 after LPS treatment.

A time × LPS interaction (*F*(3,210) = 2.8, *p* < 0.05, *ε* = 0.91) was also found for sucrose preference during the* test phase*. Post hoc analysis revealed that systemic LPS administration reduced sucrose preference close to chance level (i.e., 50%) on D1 (Figure 4(c), right panel). Interestingly, the LPS-induced decrease of sucrose preference lasted until D3, a time point at which total volume intake had returned to control levels suggesting occurrence of anhedonia in the absence of sickness on D2 and D3.

### 3.4. Repeated LPS Exposure Protects against Acute LPS-Induced Sickness but Not Anhedonia

The duration of inflammatory processes associated with depression is thought to be chronic rather than acute. In this experiment, the anhedonic response to a more prolonged immune challenge was investigated by first injecting rats with LPS on 5 consecutive days (*preexposure phase*) and measuring sucrose preference after an acute LPS injection 3 days later (*test phase*).

**Figure 5:**
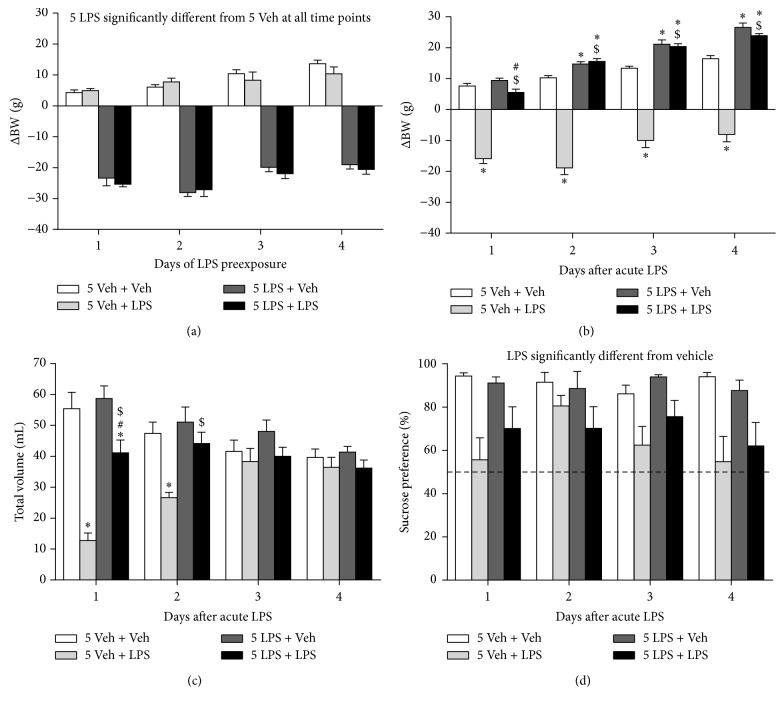
Repeated LPS administration protects against LPS-induced sickness but not anhedonia. After the* familiarization phase* (data not shown), rats received daily i.p. injections of either 0.63 mg/kg LPS (5 LPS) or vehicle (5 Veh) for 5 consecutive days. Three days after this* preexposure phase*, an acute systemic injection was administered to rats of either 0.63 mg/kg LPS or vehicle (Veh) and voluntary consumption of water and sucrose was measured during a period of 24 h for 4 days. Repeated peripheral LPS administration reduced body weight during the* preexposure phase* (a). At the beginning of the* test phase*, rats preexposed to LPS had a significant lower weight than animals that received vehicle preexposure (288.9 ± 2.6 g versus 314.5 ± 3.6 g, *p* < 0.001; data not shown). Weight only decreased mildly upon rechallenge with LPS, while weight reduction in LPS naive rats was more pronounced (b). On the first day of the* test phase*, LPS-challenged rats drank less than their vehicle-injected controls but this effect was less pronounced in rats that were preexposed to LPS (c). Sucrose preference was reduced in LPS-treated rats but no effect of preexposure was found (d). Dashed lines indicate chance level for sucrose preference. Graphs are plotted as mean + SEM (*n* = 12 per group). Data were analyzed by rmANOVA followed by independent samples *t*-test. ^*∗*^ *p* < 0.05 compared to 5 Veh + Veh, ^#^ *p* < 0.05 compared to 5 LPS + Veh, and ^$^ *p* < 0.05 compared to 5 Veh + LPS. 5 Veh + Veh: 5 days of vehicle followed by acute vehicle, 5 Veh + LPS: 5 days of vehicle followed by acute LPS, 5 LPS + Veh: 5 days of LPS followed by acute vehicle, and 5 LPS + LPS: 5 days of LPS followed by acute LPS.

The effect of repeated LPS administration on the growth rate of rats was determined by calculating the change in body weight versus their weight immediately before the first LPS injection in the* preexposure phase*. Factorial rmANOVA demonstrated a time × preexposure interaction for change in body weight during this* preexposure phase*. Post hoc analysis showed that rats receiving vehicle injections continuously grew, while rats preexposed to LPS showed reduced weight change at all days of the* preexposure phase* (Figure 5(a)).

At the beginning of the* test phase*, rats that were preexposed to LPS weighed significantly less than animals that received vehicle preexposure (i.e., 288.9 ± 2.6 g versus 314.5 ± 3.6 g, *p* < 0.001; data not shown). To evaluate the weight change after a subsequent acute LPS injection, weight measures during the* test phase* were subtracted from the weight at the start of the* test phase*. rmANOVA revealed that there was a time × preexposure × LPS interaction (*F*(3,132) = 13.9, *p* < 0.001, *ε* = 0.76) for change in body weight in the* test phase*. Post hoc analysis indicated that all rats lost weight after receiving the acute LPS challenge (Figure 5(b)). Animals that received LPS during the* preexposure phase*, however, lost significantly less weight after the acute LPS injection than rats that were pretreated with vehicle. Moreover, rats preexposed to LPS recovered faster after the acute LPS challenge than rats that received acute LPS after vehicle pretreatment.

There was a time × preexposure × LPS interaction for total volume intake during the* test phase*. All groups that received LPS in the* test phase* drank less than vehicle-injected animals on the first day after acute LPS administration (Figure 5(c)). However, LPS-pretreated rats drank much more upon a subsequent acute LPS challenge than animals that were preexposed to vehicle. On the second day after acute LPS injection, the total volume drank by rats preexposed to LPS had returned to control levels, while this took until day 3 for vehicle-pretreated rats.

Finally, factorial rmANOVA revealed that, for sucrose preference during the* test phase*, there was a main effect of LPS (*F*(1,44) = 24.4, *p* < 0.001, *ε* = 0.93) but not of time or preexposure. Rats that were injected with LPS during the* test phase* had a reduced sucrose preference when compared to vehicle-injected animals (Figure 5(d)). However, due to the absence of other main effects no further post hoc analyses could be made.

### 3.5. Subchronic Restraint Stress Does Not Influence the Anhedonic Response to a Subsequent LPS Challenge

Stress, a known risk factor for depression, influences immunological responses. To test whether stress impacts on the anhedonic response to an immune challenge, rats were first exposed to 1 h of restraint stress per day for 5 consecutive days (*manipulation phase*) and subsequently injected systemically with LPS three days later (*test phase*).

**Figure 6:**
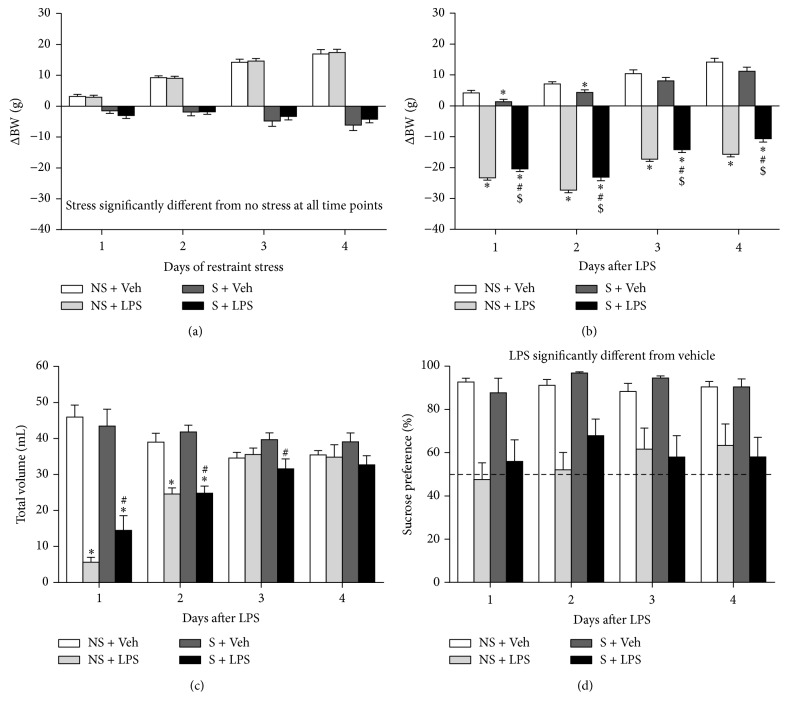
Subchronic restraint stress does not influence the anhedonic response to a subsequent LPS challenge. After the* familiarization phase* (data not shown), rats were exposed to 1 h of restraint stress daily for 5 consecutive days. Three days after the last stress session animals received an i.p. injection of either vehicle or 0.63 mg/kg LPS. Daily restraint stress reduced body weight (a). At the beginning of the* test phase*, rats that were stressed during the* manipulation phase* had a significant lower body weight than animals that were nonstressed (299.7 ± 3.1 g versus 324.9 ± 2.4 g, *p* < 0.001; data not shown). A subsequent acute LPS challenge reduced weight in nonstressed rats and to a slightly lesser extent in stressed animals (b). Systemic LPS administration also reduced total volume intake (c) and sucrose preference (d), but no differences could be found between stressed and nonstressed rats. Dashed lines indicate chance level for sucrose preference. Graphs are plotted as mean + SEM (*n* = 12 per group). Data were analyzed by rmANOVA followed by independent samples *t*-test. ^*∗*^ *p* < 0.05 compared to NS + Veh, ^#^ *p* < 0.05 compared to S + Veh, and ^$^ *p* < 0.05 compared to S + LPS. NS: nonstressed, S: stressed, and Veh: vehicle.

The effect of subchronic restraint stress on the growth rate of rats was determined by calculating the change in body weight during the* manipulation phase* versus the weight just before the first stress session. Factorial rmANOVA revealed a time × stress interaction (*F*(3,138) = 145.6, *p* < 0.001, *ε* = 0.69) for change in body weight during the* manipulation phase*. Post hoc analysis indicated that stressed rats continuously lost weight from the first stress session until the last, while nonstressed rats grew steadily during the* manipulation phase* (Figure 6(a)).

Rats that were stressed weighed significantly less than nonstressed animals at the beginning of the* test phase *(i.e., 299.7 ± 3.1 g versus 324.9 ± 2.4 g, *p* < 0.001; data not shown). Weight changes induced by a subsequent acute LPS challenge were determined by subtracting weight measures from the rats' weight at the beginning of the* test phase*. There was a time × LPS (*F*(3,132) = 23.9, *p* < 0.001, *ε* = 0.63) and a stress × LPS (*F*(1,44) = 16.0, *p* < 0.001, *ε* = 0.63) interaction for change in body weight during the* test phase*. Post hoc analysis showed that LPS decreased weight in stressed and nonstressed rats (Figure 6(b)). This LPS-induced weight loss was most pronounced in the first 2 days after administration and then recovered over time. For total volume intake during the* test phase*, there was a time × stress × LPS interaction (*F*(3,132) = 4.5, *p* < 0.01, *ε* = 0.75). On the first 2 days after administration, stressed and nonstressed rats that were injected with LPS drank significantly less than animals that received vehicle (Figure 6(c)). On the third day after LPS administration, stressed rats that received LPS drank less than their vehicle-injected controls, while the total volume intake of LPS-treated nonstressed rats had returned to control values. Finally, factorial rmANOVA indicated that there was a significant LPS effect (*F*(1,44) = 59.0, *p* < 0.001, *ε* = 0.85), but no main effect of time or stress for sucrose preference during the* test phase*. LPS-treated rats had a lower sucrose preference than vehicle-injected animals, but the lack of main effects of time and stress did not allow further post hoc analysis (Figure 6(d)).

## 4. Discussion

Anhedonia, or the inability to experience pleasure from naturally rewarding activities, is a hallmark of clinical depression. While other key symptoms such as depressed mood are challenging to measure in laboratory animals, anhedonia can be estimated fairly easy by measuring the preference an animal develops for a sweetened solution relative to water. It is suggested that a decrease in this preference reflects a state of anhedonia [19].

Systemic administration of LPS has been commonly used to study inflammation-associated depression in rodents. However, discrepancies in the doses administered and time points investigated between labs have made it difficult to establish this approach as a useful animal model to study depressive symptoms. In a previous study, we characterized LPS-induced behavioral changes in mice and demonstrated that the time course of sickness and anhedonia can be evaluated by measuring total volume intake and sucrose preference in the SPT [17]. To extend this work, we assessed the anhedonic response to LPS in rats, while paying close attention to the dose- and time-dependency of LPS-induced sickness behavior because this may confound behavioral readouts being interpreted as depressive-like symptoms.

First, LPS-induced behavioral changes were evaluated in automated, investigator-independent assays commonly used to measure sickness and depressive-like behavior in rodents. In this study the animals were naive to testing at each time point measured, thus excluding potential confounding effects caused by habituation to repeated testing at different time points. Sickness, as measured by reduced locomotor activity in the OFT, was present as soon as 2 h after LPS administration and started to dissipate at 24 h. However, total volume intake in the SPT was still decreased at this time, thereby indicating that sickness had not disappeared completely. Interestingly, sucrose preference in rats injected with the low dose of LPS (0.31 mg/kg) had returned to control levels at 24 h, despite the fact that these animals still drank much less than vehicle-treated rats. Rats injected with higher doses of LPS, in contrast, showed reduced total volume intake and decreased sucrose preference at this time. This may indicate that, at a dose of 0.63 mg/kg (and higher), LPS causes more pronounced anhedonia and therefore this particular dose was selected for further experimentation.

While the immunological response to LPS in mice and its relationship to behavioral changes are well documented, this is not the case for rats. Currently available literature on the effect of systemic LPS administration on circulating levels of inflammatory mediators in the rat is limited by the number of analytes measured and/or time points used. We have previously shown that in the mouse LPS administration leads to a rapid release of proinflammatory cytokines such as TNF-*α*, IL-1*β*, and IL-6 [17]. The present study for the first time provides the serum profile of a broad panel of immune molecules over a prolonged period of time after LPS administration. We show that systemic LPS injection in rats leads to a response comparable to that in mice, with serum levels of proinflammatory cytokines peaking between 1 and 2 h after LPS administration and returning to baseline levels at 24 h. These findings are in line with Goble et al., who previously reported rapid, but short-lasting, increases in circulating IL-1*β* and IL-6 in LPS-challenged rats [20]. In our study, the release of the anti-inflammatory cytokine IL-10 peaked at 1 h and faded in the first 24 h after LPS. This confirms that the strong inflammatory response to a peripheral immune challenge is tightly regulated and rapidly attenuated by anti-inflammatory mediators. The appetite suppressing hormone leptin, whose primary function is to regulate energy balance [21], is also known to be an important mediator of sickness during systemic inflammation [22]. In line with other studies [23, 24], we found that peripheral LPS administration increased circulating levels of leptin. Although this effect was short lasting, it is not unlikely that leptin plays a role in the reduction of fluid intake and body weight that follows after LPS injection. The LPS-induced changes in serum levels of most analytes had dissipated by 24 h. However, circulating levels of the chemokines CXCL1, MCP-1, and MIP-1*α* remained elevated up to 96 h after treatment. These chemokines play an important role in leukocyte migration and activation, and their serum profile suggests that the immunological response to systemically administered LPS lasted for several days. Future studies should reveal whether levels of circulating chemokines could be used as a biomarker for inflammation-associated depressive symptoms such as anhedonia.

Activation of the immune system is known to deregulate the HPA axis, a physiological finding which is frequently observed in depression [25]. In agreement with previous findings [20, 26], we found that systemic LPS injection increased serum levels of corticosterone. This release of corticosterone into the circulation occurred promptly after LPS administration and had decreased substantially by 24 h. Corticosterone levels also increased in vehicle-injected rats. However, this happened to a lesser extent than following LPS administration and probably occurred as a consequence of stress related to the experimental procedure. While it is clear that inflammation can induce behavioral changes through secretion of systemic mediators, the precise relationship between specific cytokines and behavioral changes is not yet well understood. Sickness behavior is a normal physiological response that evolved to help organisms cope with infections [27]. It is thus unlikely that short-lived increases in cytokines playing a role in sickness behavior (such as IL-1*β*, IL-6, and TNF-*α*) will induce depression. Nevertheless, these cytokines may cause depressive symptoms such as anhedonia and thus add to the understanding of the pathological mechanisms of depression.

In our extended SPT study we showed that a single bolus of LPS reduces fluid intake in the first 2 days after administration. More specifically, on the first day after administration, LPS decreased total volume intake to approximately one-third of the normal daily intake (i.e., unchallenged on a W/W day). This suppressed drinking is a clear indication of sickness and precludes interpreting reduced sucrose preference as a sign of anhedonia. On the second day, however, LPS-injected rats also drank less than vehicle-treated controls but their total volume intake did reach normal daily intake levels. Therefore, the reduced sucrose preference seen on this day can be interpreted as an anhedonic response to the LPS challenge. This became even clearer on the third day where there was no difference in total volume intake between treatment groups, but still a significant reduction in sucrose preference in LPS-injected rats. The fact that rats treated with LPS started to gain weight at day three further indicates that sickness had dissipated at this point. In line with previously reported rat data [28, 29], LPS administration did not alter water intake at any of the time points (data not shown). This highlights the importance of including measures of total volume intake to estimate sickness in paradigms such as the SPT.

In our model of acute systemic LPS administration, anhedonia was present in the absence of apparent sickness from 2 to 3 days after injection. Depressive episodes in humans, however, can last up to several months [30]. Moreover, inflammation-associated depression in humans develops on a background of persistent inflammation. In order to mimic the human situation more closely, we decided to evaluate the anhedonic response to a longer lasting immune challenge. It was found that preexposure to LPS reduced the sickness response to a subsequent acute LPS challenge, suggesting the induction of tolerance to LPS. This phenomenon involves downregulation of proinflammatory responses to repeated LPS exposure and is thought to protect an organism from excessive tissue damage and the development of pathological states during prolonged or uncontrolled inflammation [31, 32]. Moreover, in our study, the secondary LPS challenge reduced sucrose preference in all rats, regardless of their preexposure. This indicates that, in our model to study anhedonia, repeated LPS administration does not offer an advantage over a single peripheral injection. Kubera and coworkers recently described a model in which repeated LPS injections given at one-month intervals induced a chronic state of anhedonia in female, but not in male mice [33]. Our study was performed in male rats, which could be an explanation for the lack of effect. Additionally, it is possible that a more specific or elaborate LPS dosing scheme is required to induce more pronounced and/or longer lasting anhedonia.

Stress is a major risk factor for the development of depression [34]. In a second approach to create a model of anhedonia that relates to the human situation, we tested whether exposure to stress alters anhedonic responses to a subsequent immune challenge. It was observed that repeated daily exposure to restraint stress decreased body weight, indicating that the rats underwent stress. Previous work by other labs has shown that repeated restraint stress can induce depressive-like behavior, including anhedonia [35–39]. Moreover, in studies using social disruption as a model of psychological stress the sickness response to a secondary LPS challenge was aggravated [40, 41]. In our study, repeated restraint stress did not alter measures of sickness or anhedonia in response to a subsequent LPS challenge. These findings are in line with results from a study by Wohleb et al., where repeated social defeat stress in mice did not exacerbate anxiety behavior following a secondary LPS challenge [42]. It is possible that the restraint stress protocol used in our study was too mild and thus not sufficient to alter the sickness and/or anhedonic response to a subsequent immune challenge. Indeed, in most studies where anhedonia was reported following repeated restraint stress, the animals were restrained for several hours per day (versus 1 hour in our study), during several weeks (versus 5 days in our study) [36, 38, 39], and when shorter lasting restraint stress protocols were used, anhedonia was evaluated immediately after the last restraint session [35].

In the experiments where we tested the effect of LPS preexposure or stress on the anhedonia response to a subsequent LPS administration, LPS-injected rats showed reduced sucrose preference across the test phase. These findings are not in line with the acute LPS experiment where sucrose preference was only reduced at days 2 and 3 but had recovered at day 4. This discrepancy can potentially be explained by the fact that in our more elaborate experimental protocols a third phase was introduced between the* familiarization phase* and* test phase*. It may be possible that this extra week of individual housing and handling confounded measures of sucrose preference after a subsequent systemic LPS injection.

## 5. Conclusion

This study provides a systematic analysis of the time course of cytokine release and behavioral changes following peripheral LPS administration in rats. We report a SPT protocol that includes measurements of total volume intake, sucrose preference, and body weight and demonstrated that, by assessing these measurements and their interaction, this SPT protocol provides a way of separating LPS-induced anhedonia from sickness. This anhedonic response to LPS is robust but only lasts for 2 days. Therefore, caution is needed when studying the mechanisms underlying inflammation-associated depression using a single LPS injection in rats. To model the chronic nature of depression in humans more carefully, our SPT protocol was used to test whether preexposure to repeated LPS administration or subchronic stress influences the anhedonic response to a subsequent LPS challenge. While these procedures did not affect the time course of anhedonia, our results provide useful insights into the behavioral consequences of peripheral immune activation using LPS and may contribute to the development of more elaborate rodent models of inflammation-associated depression.
